# 核心蛋白聚糖decorin在非小细胞肺癌组织中低表达且与组织学类型相关

**DOI:** 10.3779/j.issn.1009-3419.2011.11.03

**Published:** 2011-11-20

**Authors:** 永凯 吴, 汀 肖, 敏 李, 莹 张, 燕宁 高, 克林 孙

**Affiliations:** 1 100021 北京，北京协和医学院中国医学科学院肿瘤医院胸外科 Department of Thoracic Surgery, Cancer Institute (Hospital), Peking Union Medical College and Chinese Academy of Medical Sciences, Beijing 100021, China; 2 100021 北京，北京协和医学院中国医学科学院肿瘤医院肿瘤研究所“癌发生及预防分子机理”北京市重点实验室 Beijing Key Laboratory for Carcinogenesis and Cancer Prevention, Cancer Institute (Hospital), Peking Union Medical College and Chinese Academy of Medical Sciences, Beijing 100021, China

**Keywords:** 核心蛋白聚糖, 肺肿瘤, 低表达, Decorin, Lung neoplasms, Down-regulation

## Abstract

**背景与目的:**

核心蛋白聚糖（decorin）是细胞外基质（extracellular matrix, ECM）的组成成分，参与抑制胶原纤维形成、调控细胞的增殖和粘附等过程。本研究检测非小细胞肺癌（non-small cell lung cancer, NSCLC）患者肿瘤组织中decorin的蛋白水平，并研究其作为肿瘤抑制因子与各临床指标的关系及其意义。

**方法:**

采用Western blot方法检测16例肺鳞癌患者的肿瘤组织及其配对正常肺组织中decorin蛋白水平。采用免疫组织化学染色的方法检测51例NSCLC患者的肿瘤组织和正常肺组织中decorin蛋白水平。

**结果:**

Western blot结果表明相对于癌旁肺组织，12例（75.0%）肺癌组织中decorin的蛋白表达水平明显下调。Decorin在肺癌的肿瘤组织表达阳性率仅为11.8%，明显低于正常肺泡组织（阳性率为53.1%，*P* < 0.001）。并且，decorin在肺腺癌组织中几乎不表达（阳性率为0），与肺鳞癌组织相比有统计学差异（阳性率为24.0%，*P*=0.006）。

**结论:**

NSCLC肿瘤组织中decorin蛋白水平明显降低并与病理类型相关，提示decorin的异常下调可能在肺腺癌的发生发展过程中发挥作用。

## Abstract (en)

**Background and objective:**

Decorin is a protein component of the extracellular matrix. It has been proven that decorin plaid an important role in matrix assembly. The protein is also capable of suppressing tumor cell growth. The aim of this study is to examine the protein levels of decorin in tissue samples of non-small cell lung cancer (NSCLC) patients and evaluate its potential clinical value.

**Methods:**

Decorin protein levels in tumor tissues and corresponding normal tissues from 16 cases of lung squamous cell carcinoma (SCC) were determined by Western blot analysis. Fifty-one NSCLC samples, including both tumor and normal lung tissues, were examined by immunohistochemical (IHC) staining.

**Results:**

Western blot results showed that decorin protein levels in 75.0% (12/16) of the SCC tumor tissues were lower than those of the corresponding normal lung tissues. IHC results showed that positively stained decorin was detected in only 11.8% of the tumor tissues, significantly lower than that observed in normal alveoli tissues (53.1%, *P* < 0.001). In lung adenocarcinoma (ADC), almost no expression of decorin was observed, in contrast to squamous cell carcinoma (SCC) (*P*=0.006).

**Conclusion:**

Decreased levels of decorin in NSCLC tissue samples, particularly ADC cases, indicate that decorin could play a role in lung cancer carcinogenesis.

肺癌已经成为全世界严重威胁人类生存健康的恶性肿瘤之一，其发病率和死亡率均列各种肿瘤之首。国家卫生部提供的资料显示，肺癌已成为我国第一大癌症。核心蛋白聚糖（decorin）是存在于细胞周围基质组织中的一种富含亮氨酸的低分子蛋白多糖，以核心蛋白（44 kDa）和蛋白聚糖（72 kDa-130 kDa, smear）两种形式表达于组织细胞中^[1]^。Decorin是细胞外基质（extracellular matrix, ECM）的组成成分，属于亮氨酸富集蛋白家族（small leucine-rich proteoglycan, SLRP）成员，参与抑制胶原纤维形成、调控细胞的增殖和粘附等过程^[2]^。

本实验室在前期工作中，利用新型的蛋白质组研究体系建立了肺癌相关蛋白数据库以寻找新的肺癌标志物^[3]^，其中包括decorin。近年来的研究表明decorin可以抑制多种肿瘤细胞系的生长、转移。目前有关其在非小细胞肺癌（non-small cell lung cancer, NSCLC）组织中表达的研究还比较少。本研究通过Western blot和免疫组织化学染色的方法，分析肺癌组织中decorin蛋白的表达情况，探讨该蛋白在肺癌发生发展中的意义。

## 1 材料与方法

### 1.1 研究对象及组织样品

所有临床组织样品取自1999年12月-2005年9月中国医学科学院肿瘤医院胸部肿瘤外科收治的患者。根据世界卫生组织（World Health Organization, WHO）2004年的肺癌组织学分型标准进行肺癌组织学分型，根据国际抗癌联盟（International Union Against Cancer, UICC）2002年发布的第6版肺癌TNM分期系统进行分期。所有患者术前均未接受过物理治疗或化学药物治疗。

用于Western blot检测的新鲜肿瘤组织样品取自接受手术治疗的16例肺鳞癌（squamous cell carcinoma, SCC）患者。收集手术切除的肿瘤组织及其配对的远端正常肺组织。患者男性15例，女性1例，中位年龄65岁（44岁-76岁）。Ⅰ期2例，Ⅱ期3例，Ⅲ期8例，Ⅳ期1例，无明确分期2例。高分化2例，中分化5例，低分化7例，无明确分化2例。

采用51例10%甲醛固定、石蜡包埋组织用于免疫组织化学染色。患者男性34例，女性17例，中位年龄53岁（33岁-76岁）。Ⅰ期14例，Ⅱ期17例，Ⅲ期15例，Ⅳ期3例，无明确分期2例。高分化3例，中分化23例，低分化25例。

### 1.2 组织蛋白抽提和保存

取适当大小的组织块，加入300 μL细胞裂解液（包含RIPA裂解缓冲液、蛋白酶抑制剂PSMF），置于冰上裂解30 min。裂解液于4 ℃离心20 min，12, 000 rpm。收集上清液，分装后保存于-80 ℃冰箱。采用BCA Protein Assay Kit（Pierce, IL, USA）进行蛋白定量。

### 1.3 Western blot检测组织样品中decorin蛋白水平

采用10%SDS-PAGE分离胶电泳分离变性后的40 μg组织蛋白，随后使用半干式电转仪将分离后的蛋白转移至硝酸纤维素膜，电转后的膜在5%脱脂奶粉中室温封闭1 h。膜在decorin鼠单抗溶液（1:1, 000, R & D, USA）中4 ℃孵育过夜，然后在带辣根过氧化物酶标签的羊抗鼠二抗溶液（1:4, 000, Jackson ImmunoResearch, USA）中室温孵育1 h，用发光试剂盒（Santa Cruz Biotechnology, CA, USA）检测，X线胶片曝光、显影、定影。以β-actin作为内参蛋白，使用β-actin鼠单抗（1:3, 000, Sigma-Aldrich, USA）作为第一抗体。

### 1.4 免疫组织化学染色方法

采用SP法，鼠源decorin抗体（R & D, USA）稀释度为1:200，染色操作程序参照试剂说明书进行。将石蜡切片经二甲苯、梯度乙醇脱蜡至水; 3%H_2_O_2_处理15 min，1×PBS洗3次，每次3 min; 血清封闭20 min; 甩去封闭血清，加稀释好的一抗，4 ℃过夜; 1×PBS洗3次，生物素标记的二抗，37 ℃孵育30 min; 1×PBS洗3次，加辣根过氧化物酶标记的链霉卵白素，37 ℃孵育30 min; DAB显色，苏木素复染; 切片经梯度乙醇，二甲苯脱水，中性树脂胶封片。以PBS代替一抗作为阴性对照。显微镜下观察。免疫组织化学染色图片由莱卡DFC425图像系统采集。

Decorin蛋白显示细胞浆着色，染色强度判断标准^[4]^为：无染色=0，轻度染色（浅黄色）=1，中度染色（棕黄色）=2，强染色（黄褐色）=3;染色面积判断标准为：无细胞染色=0，< 25%细胞染色=1，25%-50%细胞染色=2，> 50%细胞染色=3。两种积分相加，按照：（-）=0，（+）=1-2，（++）=3-4，（+++）=5-6。进行半定量判断，≥（++）为阳性结果，（-、+）为阴性结果。

### 1.5 统计学方法

采用SPSS 15.0软件进行统计学分析。使用非参数检验*Mann-Whitney U*检验比较免疫组织化学染色结果的组间差异。*P* < 0.05为有统计学差异。

## 2 结果

### 2.1 Western blot检测组织样品中decorin蛋白表达

Decorin以核心蛋白（44 kDa）和蛋白聚糖（72 kDa-130 kDa, smear）两种形式表达于组织细胞中。16对配对组织蛋白标本中，相对于癌旁肺组织，12例（75.0%）肺癌组织中核心蛋白形式及蛋白聚糖形式的decorin表达水平明显下调（图 1）。

**1:**
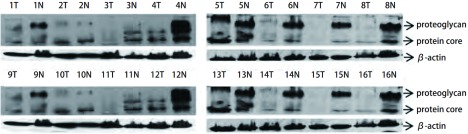
Western blot检测decorin蛋白在16对配对的肺鳞癌组织（T）及癌旁肺组织（N）中的表达 The expression of decorin protein in tumor tissues (T) and corresponding normal tissues (N) from 16 cases of lung squamous cell carcinoma by Western bolt

### 2.2 免疫组织化学染色

#### 2.2.1 Decorin蛋白在肺癌组织中的表达明显下调（表 1，图 2A，图 2B）

**1:** 非小细胞肺癌组织中decorin蛋白的表达情况 Expression of decorin in tissue samples from non-small cell lung cancer patients by immunohistochemical staining

Characteristic	*n*	Expression of decorin [*n* (%)]		*P*
		Negative	Positive	
Alveolar	49	23 (46.9)	26 (53.1)	< 0.001^a^
Bronchial	34	30 (88.2)	4 (11.8)	0.352^b^
NSCLC	51	45 (88.2)	6 (11.8)	
Histological type				0.006^c^
SCC	25	19 (76.0)	6 (24.0)	
ADC	26	26 (100.0)	0	
Stage				0.698^d^
Ⅰ+Ⅱ	31	28 (90.3)	3 (9.7)	
Ⅲ+Ⅳ	18	16 (88.9)	2 (11.1)	
Missing	2	-	-	
LNM				0.101^e^
Yes	32	28 (87.5)	4 (12.5)	
No	17	16 (94.1)	1 (5.9)	
Missing	2	-	-	
Grade				0.230^f^
Poor	25	22 (88.0)	3 (12.0)	
Moderate	23	20 (87.0)	3 (13.0)	
High	3	3 (100.0)	0	
*P* value from *Mann-Whitney U* test; ADC: adenocarcinoma; LNM: lymph node metastasis; NSCLC: non-small cell lung cancer; SCC: squamous cell carcinoma. ^a^NSCLC *vs* Alveolar; ^b^NSCLC *vs* Bronchus; ^c^ADC *vs* SCC; ^d^Stage Ⅰ+Ⅱ *vs* Ⅲ+Ⅳ; ^e^LNM yes *vs* no; ^f^Low grade *vs* middle grade *vs* high grade.				

**2:**
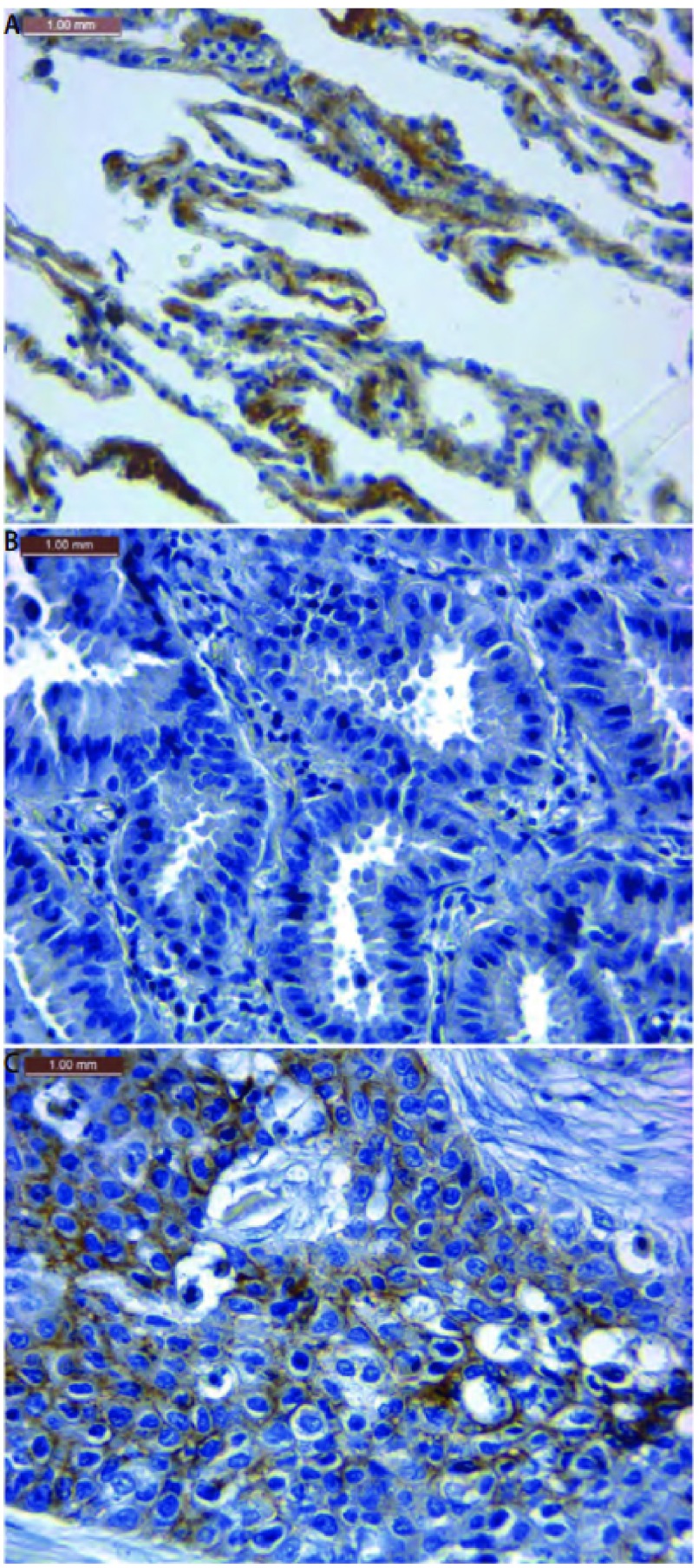
免疫组织化学法检测decorin在肺癌组织中的表达。A：Decorin在正常肺泡中表达阳性；B：Decorin在肺腺癌组织中不表达；C: Decorin在肺鳞癌组织中表达阳性。 The expression of decorin in non-small cell lung cancer tissues by immunohistochemical staining. A: The positive expression of decorin in normal alveolar; B: The negative expression of decorin in adenocarcinoma; C: The positive expression of decorin in squamous cell carcinoma.

Decorin在肺癌的肿瘤组织表达阳性率仅为11.8%（6/51），明显低于正常肺组织（阳性率为53.1%，*P* < 0.001）。Decorin在正常支气管中表达的阳性率也很低，为11.8%（4/34）。在高、中、低分化的肺癌组织中decorin的阳性率分别为：12.0%（3/25）、13.0%（3/23）、0（0/3），各组间无统计学差异（*P*=0.230）。Decorin在肺癌早期（Ⅰ期+Ⅱ期）组织中的阳性率为9.7%（3/31），晚期（Ⅲ期+Ⅳ期）为11.1%（2/18），两组之间无统计学差异（*P*=0.698）。Decorin在有淋巴结转移的肺癌组织中表达阳性率为12.5%（4/32），无淋巴结转移的肺癌组织中表达阳性率为5.9%（1/17），两组之间无统计学差异（*P*=0.101）。

#### 2.2.2 Decorin蛋白在肺癌组织中的表达与组织学类型相关（表 1）

Decorin在26例肺腺癌组织中几乎不表达，阳性率为0（0/26），而在肺鳞癌组织中阳性率为24.0%（6/25），两组之间存在明显差异（*P*=0.006，图 2C）。

## 3 讨论

Decorin是组织间联系的组成成分之一，与Ⅰ型胶原纤维结合，在基质组装中发挥重要作用。Decorin可与细胞外基质中的其他分子相互作用，影响细胞间粘附及迁移，可能是肿瘤的发生发展及转移的重要抑制因子。外源decorin可通过激活表皮生长因子受体（epidermal growth factor receptor, EGFR），调节细胞周期蛋白p21通路引起前列腺癌细胞的G_1_-S阻滞，抑制细胞生长。并且，decorin可以和TGF-β等多种生长因子结合，负性调节肿瘤细胞生长^[5]^。

Banerjee等^[6]^用免疫组化的方法研究发现，在口腔上皮的不典型增生细胞中decorin表达缺失，但在癌组织肿瘤细胞胞核异常表达，并认为这种异位表达使decorin失去了通过与TGF-β结合而阻断EGFR通路的能力。Augoff等^[2]^结果显示，相比于正常肠粘膜decorin在腺瘤中的蛋白表达发生了下降，在常见类型的肠癌组织中表达最弱。这两位学者的研究提示decorin表达的降低可能是口腔癌和结直肠癌的早期事件，并且是肿瘤发生的促进因素。本研究的Western bolt结果显示，相对于癌旁正常肺组织，核心蛋白和蛋白聚糖两种表达形式的decorin在肺鳞癌组织的蛋白含量均明显降低。我们采用免疫组织化学染色的方法，以进一步明确decorin在肺癌组织中各种细胞（如正常肺泡、正常支气管、肿瘤等）的表达情况。结果也表明，肺癌组织中的decorin表达降低，并在不同的病理组织学类型的肺癌中表达阳性率不同。在肺腺癌组织中，decorin几乎不表达，阳性率为0。与其对照的正常肺泡中，表达阳性率为53.1%，二者存在明显的统计学差异（*P* < 0.001）。而在肺鳞癌组织中，decorin蛋白表达的阳性率仅为24.0%。但是，decorin在正常支气管中表达的阳性率更低，为11.8%。此结果提示，decorin表达的降低可能不是肺癌发生的早期阶段，而在不同病理类型的NSCLC发生发展中发挥的作用不同，有可能在肺腺癌的发生中扮演重要角色。

以腺病毒载体转染decorin可抑制乳腺癌细胞系MTLn3的体内成瘤和肺部转移^[7]^。已有研究^[8-10]^显示*DCN*基因在胰腺癌、结肠癌、乳腺癌等恶性肿瘤中为低表达，*Cox*分析进一步证明decorin是乳腺癌复发及预后不良的独立预后因素。Biaoxue等^[11]^在肺癌的研究结果表明，正常肺组织中的decorin表达高于肿瘤组织，并且decorin的低表达与肺癌的不良预后和淋巴结转移明显相关。Goldoni等^[12]^的研究认为，decorin是强有效的抑制乳腺癌原发灶生长和转移的靶向治疗药物，其机制为抑制ErbB受体酪氨酸激酶的活性。而在本研究中，decorin的低表达与肺癌的淋巴结转移无关（*P* < 0.05），可能因为实验所用的病例数较少。在后续研究中应扩大病例，进一步探讨decorin与肺癌转移的相关性。
